# Supplementary material for: How does digital technology affect manufacturing upgrading? Theory and evidence from China
Source: PLoS One. 2022 May 12;17(5):e0267299. doi: 10.1371/journal.pone.0267299 (PMC9098021; doi:10.1371/journal.pone.0267299)
Supplement: Attachment — Submitted filename: review-PONE-D-21-40013.docx [file pone.0267299.s002.docx]

Relevance. The paper investigates the theoretical mechanism of digital technology for manufacturing upgrading. This paper incorporates digital technology and the global value chain division of labor into a unified analytical framework. On the one hand, digital technology's contribution is evaluated from domestic and foreign perspectives; on the other hand, the heterogeneity of manufacturing factor intensity and the classification of technology levels are used to deepen the understanding and elaboration of the relationship between digital technology for manufacturing upgrading. The research in this paper is enlightening and guiding researchers to deepen their understanding of the impact of the digital economy.

Rigour. The econometric model in this paper is reasonably and scientifically constructed and can adequately support the research topic of this paper. However, the following improvements in the research discussion need to be considered.

(1) Some expressions are inappropriate and even confusing. For example, there is a problem with the expression "1% high" in the third line of the text on page 15 of the article. Please explain the meaning of 1% in detail.

(2) Please further concise and highlight the semantic precision in the presentation of the contribution of the article.

(3) Although the data sources are explained in this paper, digitalization in China is developing rapidly. Therefore, the use of data in this paper seems to be old and the research data is rather lagging, please consider adding the latest data after 2014.

(4) Figure 1 shows the digital technology level index of China's manufacturing industry from 2000 to 2014, but the analysis of the interpretation of Figure 1 is not sufficient, and some rich descriptions expressed by the columns in the figure should be given.

(5) The text display of Table 1and Table 2 is further optimized. it currently feels like there is a problem with the typography.

Impact: This work has the potential to have a notable impact as it presents the theoretical mechanism of digital technology for manufacturing upgrading and gives the results and description of the empirical analysis.

Quality of presentation and writing. The paper followed a structure, and the quality of written communication was generally good. However, some improvements still need to be made. The authors need to optimize further the presentation of the article in terms of the various sections such as the abstract, introduction, model, empirical evidence, discussion, and especially the illustration of the content of the figures and tables. Very importantly, the authors could consider native English speakers to improve the quality of the language presentation of this article. Currently, the potential value of the article is obscured due to language presentation issues.

I am recommending that this paper be revised and re-reviewed.
