# Supplementary material for: How does digital technology affect manufacturing upgrading? Theory and evidence from China
Source: PLoS One. 2022 May 12;17(5):e0267299. doi: 10.1371/journal.pone.0267299 (PMC9098021; doi:10.1371/journal.pone.0267299)
Supplement: Attachment — Submitted filename: Response to Reviewers.docx [file pone.0267299.s003.docx]

**Response to Reviewers**

*I sincerely thank the editor and all reviewers for the valuable feedback that I have used to improve the quality of my manuscript.I have made extensive corrections to the previous paper according to your nice suggestions. I`ll be happy to edit paper further,based on helpful comments from the reviewers.The detailed corrections are listed below.*

1. Please ensure that your manuscript meets PLOS ONE's style requirements, including those for file naming.

*Response:I have revised article according to the style requirements and submitted the 3 files.*

2. We note you have included a table to which you do not refer in the text of your manuscript. Please ensure that you refer to Table 4 in your text; if accepted, production will need this reference to link the reader to the Table.

*Response:I tried to explain Quantile regression results,now revised in the paper.*

3. Please include a copy of Table 9 which you refer to in your text.

*Response:I have corrected “Table 9” to “Table 5”*

Additional Editor Comments:
1) The first part of the article is written without any references.

*Response:The first part add 7 references now.*

2) The novelty of this manuscript should be highlighted compared to the literature review.
*Response:Compared with the current research,the novelty was highlighted in the paper.*

3) All formulations need references, unless they were extracted by authors.
*Response:References have been added and marked.*

4) What is the reason for reporting data in Figure 1 until 2014? What are data for recent years?
*Response:The paper uses the data of WIOD2016 which covering the year from 2000 to 2014.Currently the data is from major transnational input-output databases include WIOD, TiVA, GTAP-ICIO and ADB-MRIO, which cover different countries, industrial sectors and time spans (see following table).*

| *Database* | *Issuing agency* | *Covered*  *Country or region* | *Covered*  *sector* | *Time* |
| --- | --- | --- | --- | --- |
| *WIOD2013* | *EU* | *40* | *35* | *1995-2011* |
| *WIOD2016* | *EU* | *43* | *56* | *2000-2014* |
| *WTO-OECD TiVA* | *WTO* | *61* | *34* | *1995,2000,2005,2008-2011* |
| *GTAP-ICIO* | *Purdue University* | *121* | *43* | *2004、2007、2011* |
| *ADB-MRIO* | *ADB* | *63* | *35* | *2000，2007-2019* |

*The world input-output database (WIOD) has been updated to 2014. The latest version of WIOD (2016) contains continuous time series data from 2000 to 2014, covering 43 countries / regions and 56 industry categories, and includes statistical data of environmental and socio-economic accounts. The total GDP of countries and regions accounts for more than 80% of the world, which can objectively reflect major global economic activities, It is widely used by the international research scholars because of its continuity and authority.*

*I added data of 2016-2019 in the first section in order to illustrate the current situation of China's digital economy.*

5)Data in Table 1 is unclear. Numbers are not in a good format. Check it again please. Such a problem could be seen for other tables.
 *Response:The format of Table 1 and Table 2 have been revised.*

6) R2 values are so low in Table 2. What is the reason? They could not be used for a correct analysis. Such a problem could be seen in some other cases in other tables. Best fitting should be bolded.
 *Response:The main objective of empirical test using panel data is to determine which explanatory variables are significant, test whether the impact of DIG on GVC-PO is significantly correlated, and the value of R-squared will not affect the interpretation of the relationship between DIG and GVC-PO. The R^2^ value is relatively small when the explanatory variable is not added as the control variable, After the explanatory variable is added, the R^2^ value becomes larger, indicating that the model interpretation is acceptable.*

*Similar situations have been encountered in the literatures such as follows:the R^2^ is 0.01 or 0.167981*

*Source：Antras P.,Chor D.,Fally T.,Hillberry R.Measuring the Upstreamness of Production and Trade Flows[J].American Economic Review,2012,102(3),412～416.*

*Source：Wang PeiZhi, Muhammad Ramzan.Do corporate governance structure and capital structure matter for the performance of the firms? An empirical testing with the contemplation of outliers[J].Plos one, February 27, 2020 https://doi.org/10.1371/journal.pone.0229157*

7) The number of references should be extended for a better discussion. Obtained results should be compared to other results of other researches.
 *Response:The number of references have been extended from 24 to 39,Obtained results had been compared to other results of other researches in the paper now.*

Reviewer #1:

—There were some errors in sentence structure and punctuation, as well as problems with table formatting. The language of the manuscript still needs improvement. In order to truly contribute to a valuable article, the author should explore related field research more deeply, and put forward the profound thoughts of contribution and innovation of this paper compared with the current research status in the first and second parts of the article. Therefore, I think the author needs to do more work, especially to revise and improve the manuscript.
 *Response:Thank you for your careful review. I have improved the language and compared with the current research in the first and second parts of the article*

—The last part of the introduction should be a summary of the manuscript research, which is a clear summary of what will be studied. It mainly restates the key steps and main ideas of the paper and shows how they are proved. I believe this will greatly increase the degree of future citation of this literature in related fields.
 *Response:The last part of the introduction restated the key steps and main ideas of the paper and showed how they are proved.*

—Although the author has made some theoretical explorations in the third section, in my opinion, the analysis in this part is not in-depth enough. The author should consider the deeper mechanism of digital technology to promote the upgrading of manufacturing industry, and make this part more theoretical support. In addition, I believe that if the author can change the research hypothesis and put forward the statement, it will make readers understand the relevant research content more clearly.
 *Response:In the third section,I tried deeper mechanism and changed the research hypothesis and put forward the statement.*

—The manuscript has made abundant robustness verification for the regression of the benchmark model and has also carried out heterogeneity analysis from different angles, which is worthy of affirmation. However, some theoretical analysis of the results is not thorough enough, especially in the case of insignificant regression results. In my opinion, detailed, reasonable and in-depth exploration can add depth to the article and explore the development of related fields.
 *Response:I tried thorough analysis,especially in the case of insignificant regression results in paper now.*

—The conclusions and suggestions are short, and the suggestions are not targeted enough .I think if this point can be improved, the research will be more oriented.

*Response:I revised conclusions and suggestions in the paper.*

Reviewer #2

(1) Some expressions are inappropriate and even confusing. For example, there is a problem with the expression "1% high" in the third line of the text on page 15 of the article. Please explain the meaning of 1% in detail.
*Response:Thank you for the suggestions.***, **, and * indicate significant at the 1%, 5%, and 10% levels respectively. All the tables are the same.Now I revised as follows:”the impact of Foreign Direct Investment(FDI) on the status of the division of labor in the manufacturing industry is negative, which is significant at the 1% level” on page 15 mentioned.*

(2) Please further concise and highlight the semantic precision in the presentation of the contribution of the article.
 *Response:I tried to highlight the contribution of the article in the paper now.*

(3) Although the data sources are explained in this paper, digitalization in China is developing rapidly. Therefore, the use of data in this paper seems to be old and the research data is rather lagging, please consider adding the latest data after 2014.
 *Response:I added the latest data in the first section.*

(4) Figure 1 shows the digital technology level index of China's manufacturing industry from 2000 to 2014, but the analysis of the interpretation of Figure 1 is not sufficient, and some rich descriptions expressed by the columns in the figure should be given.
 *Response:I further explained and illustrated Figure 1 and descriptions expressed by columns in the paper now.*

(5) The text display of Table 1and Table 2 is further optimized. it currently feels like there is a problem with the typography.
 *Response:Table 1 and Table 2 is optimized.*
