# Supplementary material for: How does digital technology affect manufacturing upgrading? Theory and evidence from China
Source: PLoS One. 2022 May 12;17(5):e0267299. doi: 10.1371/journal.pone.0267299 (PMC9098021; doi:10.1371/journal.pone.0267299)
Supplement: Attachment — Submitted filename: Response to Reviewers.docx [file pone.0267299.s004.docx]

**Response to Reviewers**

*I sincerely thank the editor and reviewers for the valuable feedback that I have used to improve the quality of my manuscript. I have made corrections to the previous paper according to your nice suggestions. Enclosed please find the responses to the reviewers. I sincerely hope this manuscript will be finally acceptable to be published. Thank you very much for all your help and looking forward to hearing from you soon.*

1. Formulations have no references. Before the formulation, at the end part of the last sentence (before the formulation), references should be mentioned.

*Response: Thanks for the suggestion. References have been mentioned and added from 39 to 51 and marked.*

2. It is better to add a quantitative result in the abstract

*Response: Thanks for the suggestion. I`ve added quantitative results in the abstract.*

3. The title of the third part in the manuscript should be shortened. It is too lengthy.

*Response: Thanks for the suggestion. It was shortened.*

Reviewer #1:

1.There are some errors in sentence structure and punctuation in parts of the manuscript, and parts of the manuscript are repeated. Authors should carefully examine the grammatical structure and make further modifications to the language of the manuscript.

*Response: Thank you for your suggestion. The spelling,punctuation errors, repeated parts have been checked and corrected.*

2.Literature Review in Part II,the author discusses the impact of digital technology on manufacturing upgrades from the definition, theoretical and empirical perspectives of digital technology. This approach is worthy of recognition. However, the authors define digital techniques in the manuscript. In the following micro-level research, I think the author should be highly integrated with the definition given by himself.
 *Response: In the micro-level research, I have integrated with the definition. I defined digital technology in the fourth part now.*

3.Although the author has done some theoretical exploration in the third part, the analysis in this part is not deep enough in my opinion. The author proposes three effect mechanisms of digital technology on the manufacturing value chain, but how are these three effects proposed, and whether there is some connection between the three. In this regard, the authors should conduct further exploration.

*Response:I made further exploration with the figure 1 to explain three effect mechanisms of digital technology on the manufacturing value chain.*

4. The manuscript has made abundant robustness verification for the regression of the benchmark model and has also carried out heterogeneity analysis from different angles, which is worthy of affirmation. However, the author's previous statement mentioned the current situation of unbalanced development of China's manufacturing regions, whether it is possible to consider the differences between regions in the heterogeneity analysis.

*Response: Thanks for your good suggestion. I gave further explaining about the differences between regions. Due to the limitation of data, the heterogeneity analysis of regional differences cannot be carried out. I hope to use new data to do further research in the future paper.*
