# Supplementary material for: How does digital technology affect manufacturing upgrading? Theory and evidence from China
Source: PLoS One. 2022 May 12;17(5):e0267299. doi: 10.1371/journal.pone.0267299 (PMC9098021; doi:10.1371/journal.pone.0267299)
Supplement: Attachment — Submitted filename: Response to Reviewers3.docx [file pone.0267299.s005.docx]

**Response to Reviewers**

*I sincerely thank editor and reviewer for the valuable feedback that I have used to improve quality of my manuscript. I have made corrections to previous paper once more. Enclosed please find the responses to reviewers. I sincerely hope manuscript will be acceptable soon.*

1) The abbreviation WIOD should be defined at first mentioning.

*Response: Thanks for the suggestion. World Input-Output Database abbreviation WIOD was defined at first mentioning now.*

2) No quantitative results could be found in the abstract.

*Response: Thanks for the suggestion. I added quantitative results in the abstract.*

3) The title of parts is lengthy. They should be shortened.

*Response: Thanks for the suggestion. Title was shortened.*

4) All formulations need references, unless they were extracted by authors.

*Response: Thanks for the suggestion. References have been mentioned and added.*

5) The conclusion part is lengthy. It should be also shortened.

*Response: Thanks for the suggestion. Conclusion part was shortened.*

Reviewer #1:

1.In terms of language expression, I think the author should pay attention to his own way of expression. In the manuscript, some expressions are inappropriate, and the semantic precision should be highlighted.

*Response: Thank you for your suggestion. The expressions were checked and corrected, the semantic precision was highlighted.*

2. In the second part of the literature review, the author has sorted out a very good framework, which is very worthy of recognition from the perspectives of definition, theory and evidence.However, the author should sort out more cutting-edge literature in related fields (such as citing literature in the past three years many times). In this manuscript, some of the literature is relatively old.

*Response: Thank you for your suggestion.The older literatures are deleted and the latest references are added.*

3. In the fourth part, the author lists China's manufacturing digital technology level index, whether it is possible to consider adding updated data.

*Response: Thanks for your good suggestion. Since the digital technology level index of China's manufacturing industry is calculated according to the World Input-Output Database, and data in the database is from year 2000 to 2014. As a remedy,updated data about digital economy was added in the first part. It is hoped to do further research in the future.*
